# Ultrastructural pathology of oligodendrocytes adjacent to microglia in prefrontal white matter in schizophrenia

**DOI:** 10.1038/s41537-018-0068-2

**Published:** 2018-12-13

**Authors:** Natalya A. Uranova, Olga V. Vikhreva, Valentina I. Rakhmanova, Diana D. Orlovskaya

**Affiliations:** Mental Health Research Centre, Zagorodnoe shosse 2, 117152 Moscow, Russia

## Abstract

Microglial activation has been proposed to be involved in the pathophysiology of schizophrenia (SCZ). We hypothesized that dystrophic alterations of oligodendrocytes previously reported in the prefrontal white matter in SCZ might be associated with microglial activation in the acute state of SCZ. White matter of the prefrontal cortex (BA10) was studied in post-mortem brain tissue from 21 SCZ cases and 20 normal controls. The SCZ group included 12 subjects with predominantly positive symptoms and 9 subjects with predominantly negative symptoms. Electron microscopy was applied to estimate cell density, size, volume fraction (Vv) and the number (*N*) of organelles in oligodendrocytes adjacent to microglia and in oligodendrocytes adjacent to myelin, neurons and capillaries and not adjacent to microglia. Cell density of oligodendrocytes was not changed in the SCZ group as compared to controls. Vv and *N* of mitochondria were significantly decreased, while Vv of vacuoles of endoplasmic reticulum and lipofuscin granules were significantly increased in oligodendrocytes adjacent to either microglia or myelin in the SCZ group and in patients displaying predominantly positive symptoms as compared to the control group. There were no significant differences between oligodendrocytes adjacent to microglia and to myelin. Vv and *N* of lipofuscin were also increased in peri-capillary oligodendrocytes. There was no effect of clinical subgroups on the parameters of peri-capillary and peri-neuronal oligodendrocytes. Though many ameboid and dystrophic microglia adjacent to oligodendrocytes were found in the SCZ samples, we provide no quantitative evidence that oligodendrocyte dystrophy is associated with microglial activation in white matter in SCZ.

## Introduction

Neuroimaging studies of patients with schizophrenia (SCZ) have provided evidence for dysconnectivity between different brain areas and a widespread disruption of white matter (WM) integrity associated with both negative and positive symptoms, as well as with cognitive disturbances.^1^ Pathology of oligodendrocytes and myelin is believed to be a biological basis for structural and functional dysconnectivity in SCZ.^1–4^ Post-mortem studies have shown an altered expression of myelin and oligodendrocyte-related genes,^2,3^ deficits of oligodendrocytes in the prefrontal^5,6^ and the anterior cingulate WM.^7^ However, other authors did not find changes in oligodendrocyte density in different WM regions in SCZ brain tissue as compared to normal controls.^8,9^ Moreover, Bernstein et al.^10^ detected an increased density of DISC1-immunoreactive oligodendrocytes in fronto-parietal WM of patients with paranoid SCZ as compared to controls and undifferentiated/residual SCZ.

The mechanisms of oligodendrocyte abnormalities both in gray matter and in WM remain unclear. Oligodendrocytes are often located adjacent to microglial cells in gray and WM in both control and SCZ cases. Qualitative ultrastructural analysis from an electron microscopic study revealed “activated” microglia, containing invaginated nuclei and vacuolated cytoplasm, adjacent to dystrophic oligodendroglia in the prefrontal WM^11–13^ and hippocampus^14^ of subjects with SCZ as compared to normal controls. These data suggest that microglial activation might be involved in oligodendrocyte abnormalities in SCZ.

Accumulating data support the notion that neuroinflammation is associated with WM pathology in patients with SCZ, contributing to structural and functional dysconnectivity.^2–4^ Post-mortem studies^15–17^ have reported some evidence for microglial activation in WM in SCZ. However, neuroimaging studies^18–20^ provide no evidence for microglial activation in WM in SCZ. Increased expression of genes related to immune and chaperone function have been reported in the prefrontal cortex in SCZ.^21^ Wierzba-Bobrowicz et al.^22,23^ have reported degeneration of microglial cells in frontal and temporal lobes in SCZ. Interestingly, a qualitative assessment of microglial morphology detected numerous activated microglial cells, identified by their ameboid morphology, in three SCZ cases, but not in controls.^9^ Using human leukocyte antibody, Fillman et al.^17^ have found a 9% increase in microglial density and a significant positive correlation between microglial density and interleukin-1β mRNA expression in dorsolateral prefrontal WM in the SCZ subjects. The observed heterogeneity of microglial reactivity in SCZ not only highlights differences in methodological approach but may also be suggestive of clinical heterogeneity. A number of studies have found associations between the magnitude of microglia ligand binding and SCZ symptom severity.^24,25^ Busse et al.^26^ have reported that there was a significant difference in microglial number between cases with paranoid versus residual SCZ. Evidence for interconnections between oligodendrocytes and microglia showed that there is a delicate balance between activated microglia being harmful for oligodendrocytes on the one hand, and on the other being necessary for their repair and genesis. Oligodendrocytes, in turn, can control microglial activity through the production of chemokine, cytokines, and chaperokines.^27^ Previously^28^ we have reported a significant increase in areas of the nucleus and cytoplasm of microglial cells adjacent to oligodendrocytes in the prefrontal WM in the subgroup of SCZ subjects with predominantly positive symptoms as compared to controls. However, the ultrastructural parameters of oligodendrocytes were not measured. We hypothesized that dystrophic alterations of oligodendrocytes might be associated with microglial activation in the acute state of SCZ, and thus oligodendrocyte alterations might be more prominent in the subgroup of patients with predominantly positive symptoms than in the subgroup with predominantly negative symptoms (SPNS).

We aimed to perform an electron microscopic morphometric study of oligodendrocytes adjacent to microglia and of oligodendrocytes adjacent to myelin, neurons, and capillaries that were not adjacent to microglia in WM of the prefrontal cortex in the subgroup with predominantly positive symptoms (SPPS), the SPNS, and in normal controls.

## Results

**Fig. 1:**
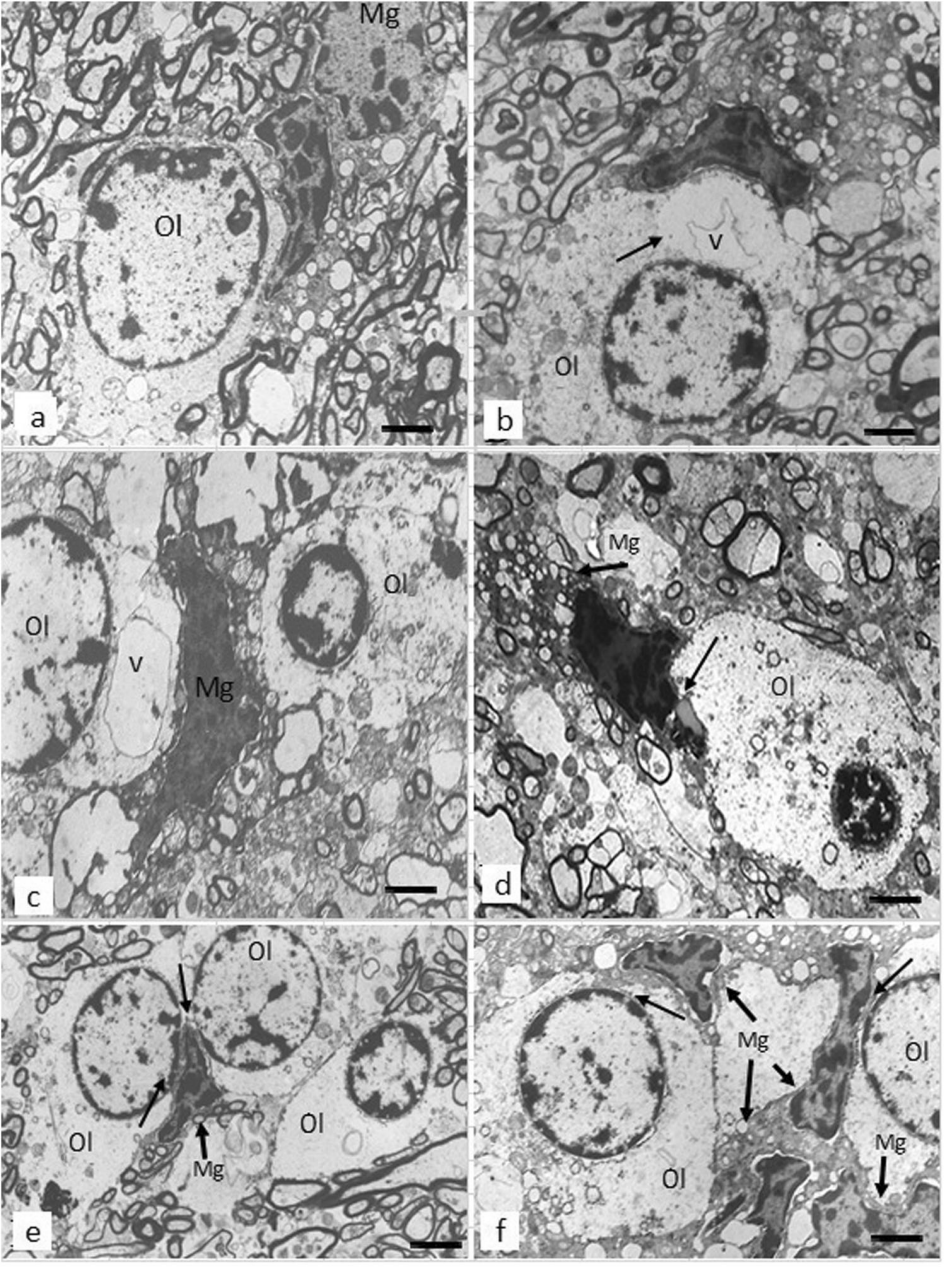
These micrographs from the prefrontal white matter show normal oligodendrocyte **a** and dystrophic changes of oligodendrocytes adjacent to microglial cells in SCZ brains **b**–**f**. Focal lysis of cytoplasm with the formation of vacuole in oligodendrocyte adjacent **b** to ameboid microglial cell and **c** to dystrophic microglial cell; **d** dystrophic microglial cell adjacent to lipofuscin granule in oligodendrocyte (arrow); **e**, **f** clusters of oligodendrocytes adjacent to microglial cell **e** and to microglial cell clusters **f**; **e**, **f** a contact of cytoplasm of microglial cells with the oligodendrocyte nucleus (arrows). Ol oligodendrocyte, Mg microglia, v vacuole (scale bar = 1 μm)

Demographic and clinical data are given in Table 1. Microglial cells in both control and SCZ subjects included different ultrastructural types. Ramified (“resting”) microglia contained relatively small somas while the amoeboid (activated) microglial cells showed cytoplasmic hypertrophy, irregular contours of vacuolated cytoplasm, and nucleus without cellular orientation. Dystrophic microglia exhibited a dark, electron-dense nucleus and vacuolated cytoplasm. Rod-shaped microglia exhibited elongated sausage-like soma representing activated microglia formed by migrating and proliferating microglia.^29^ The rod-like microglial cells were often present in both control and SCZ samples. Intermediate subtypes of microglia were also present in both groups studied. Microglial cells were often located adjacent to oligodendrocytes and touched these cells, suggesting direct contact of microglia with oligodendrocytes (Fig. 1).

**Table 1:** Demographic and clinical data (means ± standard deviation)

Subjects	Number per group	Gender^a^	Age (years)^b^	PMI (hours)^c^	Duration of disease (years)	Age of onset of disease ≤21 (years)	Age of onset of disease >21 (years)	NTR (chlorpromazine equivalents)
Controls	20	11 M/9 F	55.9±13.8	6.0±0.9				
Schizophrenia	21	10 M/11 F	56.4±17.2	6.6±1.8	26.7±0.8	15.0±2.5	33.20±9.3	416.9±29.8
SPNS	9	4 M/5 F	59.78±18.7	5.83±1.0	31.78±12.9	16.67±2.1	33.83±9.4	487.4±35.6
SPPS	12	5 M/7 F	53.92±16.4	7.13±2.1	26.00±12.2	13.33±1.5	32.78±9.7	360.5±23.5

*SPPS* the subgroup with predominantly positive symptoms, *SPNS* the subgroup with predominantly negative symptoms, *NTR* neuroleptic treatment. Controls vs. SCZ: ^a^ Chi-square test (*p* = 0.43), ^b^ANOVA (*p* = 0.92), ^c^ANOVA (*p*  = 0.24)

Oligodendrocytes adjacent to microglial cells and to myelinated fibers in subjects with SCZ looked swollen and vacuolated as compared to controls, showing the paucity of ribosomes and accumulation of lipofuscin granules (Fig. 1). On the contrary, peri-neuronal and peri-capillary oligodendrocytes looked normal in the SCZ samples as compared to controls. No signs of degeneration of oligodendrocytes were found in WM in either SCZ or control brains. Many microglial cells in the SCZ samples located in close apposition to oligodendrocytes were ameboid (Fig. 1b–f) or dystrophic (Fig. 1c, d). Focal lysis of cytoplasm of oligodendrocytes with the formation of large vacuoles was often seen in SCZ but not in controls (Fig. 1b, c). Clusters of oligodendrocytes and microglial cells adjacent to each other were seen in three SCZ cases and in one control case. In cell clusters, the cytoplasmic membrane of microglial cells directly contacted the nucleus of oligodendrocytes (Fig. 1e, f). Microglia in these clusters looked ameboid (Fig. 1f). No signs of microglia degeneration (signs of apoptotic changes) were seen in both control and SCZ samples.

The effect of diagnosis was found on volume fraction (Vv) and the number (*N*) of mitochondria, vacuoles of endoplasmic reticulum, and lipofuscin granules in oligodendrocytes adjacent to microglia (Table 2). A significant decrease in Vv (40%, *p* < 0.01) and *N* of mitochondria (35%, *p* < 0.05) and a significant increase in Vv and *N* of vacuoles (100%, *p* < 0.05) and Vv and *N* of lipofuscin granules (200%, *p* < 0.001) were detected in the SCZ group as compared to the control group (ANCOVA). Also, a significant effect of diagnosis was found on Vv and *N* of mitochondria, vacuoles, and lipofuscin granules in oligodendrocytes adjacent to myelinated fibers (Table 2). A significant decrease in Vv (38%, *p* < 0.01) and *N* of mitochondria (34%, *p* < 0.01), an increase in Vv of vacuoles (68%, *p* < 0.05) and *N* of vacuoles (136%, *p* < 0.01) as well as in Vv and *N* of lipofuscin granules (132%, 140%, *p* < 0.01) were revealed in the SCZ group as compared to the control group (ANCOVA). The effect of diagnosis was also found on Vv and *N* of lipofuscin granules in peri-capillary oligodendrocytes (Table 2). Vv and *N* of lipofuscin granules increased (200%, 260%, respectively, *p* < 0.05) in peri-capillary oligodendrocytes in SCZ as compared to controls (ANCOVA). No effect of diagnosis was revealed on the parameters of peri-neuronal oligodendrocytes (Table 2). No effects of diagnosis were found on cell density of oligodendrocytes adjacent to microglial cells, myelinated fibers, neurons, and capillaries (Table 2). Effect size for the parameters of oligodendrocytes adjacent to microglia and to myelin was moderate or large (Table 2).

**Table 2:** Effect of diagnosis on oligodendrocytes adjacent to microglia and myelinated fibers

	Oligodendrocytes adjacent to microglia				Oligodendrocytes adjacent to myelinated fibers			
	Controls (*n*=20)	SCZ (*n*=21)	F(1,37)	*p*-value/Cohen’s *d*	Controls (*n*=20)	SCZ (*n*=21)	F(1,37)	*p*-value/Cohen’s *d*
Area of oligodendrocyte (μm^2^)	61.51±2.6	63.57±2.8	0.40	0.53/0.16	63.43±2.4	65.05±2.2	0.19	0.67/0.15
Area of nucleus (μm^2^)	18.16±1.0	18.44±1.0	0.08	0.76/0.1	20.08±0.7	21.04±0.8	0.67	0.42/0.27
Area of cytoplasm (μm^2^)	43.35±2.0	45.13±2.1	0.51	0.48/0.19	43.34±1.9	44.01±1.6	0.04	0.83/0.1
Nucleus/cytoplasm ratio	0.43±0.0	0.41±0.0	0.26	0.61/0.16	0.47±0.0	0.48±0.0	0.31	0.58/0.17
Area of mitochondria (μm^2^)	0.51±0.0	0.5±0.0	0.15	0.70/0.19	0.53±0.0	0.55±0.1	0.47	0.50/0.12
Vv of mitochondria (%)	4.53±0.5	2.73±0.4	7.93	<0.01**/0.83	4.21±0.3	2.61±0.4	9.56	<0.01**/0.91
*N* of mitochondria	3.7±0.3	2.43±0.4	6.58	0.02*/0.76	3.42±0.3	2.26±0.3	6.69	0.01*/0.79
Area of vacuoles (μm^2^)	1.38±0.3	1.47±0.3	0.07	0.80/0.1	1.67±0.3	1.3±0.2	0.79	0.38/0.38
Vv of vacuoles (%)	0.92±0.2	1.87±0.3	6.52	0.02*/0.73	0.77±0.2	1.3±0.2	4.37	0.04*/0.58
*N* of vacuoles	0.35±0.1	0.76±0.2	6.12	0.02*/0.70	0.22±0.0	0.52±0.1	7.98	<0.01**/0.79
Area of lipofuscin granules (μm^2^)	1.62±0.3	1.64±0.2	0.00	0.95/0.01	1.89±0.4	1.83±0.2	0.10	0.75/0.05
Vv of lipofuscin granules (%)	0.64±0.2	1.91±0.3	17.27	<0.01***/0.99	0.77±0.2	1.79±0.3	10.7	<0.01**/0.81
*N* of lipofuscin granules	0.19±0.0	0.56±0.1	15.36	<0.01***/1.01	0.19±0.0	0.49±0.1	9.38	<0.01**/0.77
Cell density (N/mm^2^)	69.04±9.9	59.93±6.2	0.79	0.38/0.24	144.2±22.0	100.8±13.6	2.92	0.10/0.51
	Peri-neuronal oligodendrocytes				Peri-capillary oligodendrocytes			
	Controls (*n*=15)	SCZ (*n*=16)	F(1,27)	*p*-value/Cohen’s *d*	Controls (*n*=19)	SCZ (*n*=20)	F(1,35)	*p*-value/Cohen’s *d*
Area of oligodendrocyte (μm^2^)	57.69±3.2	58.16±2.8	0.08	0.78/0.04	55.46±2.9	57.53±2.9	0.18	0.68/0.16
Area of nucleus (μm^2^)	18.19±1.2	19.07±1.4	0.29	0.59/0.17	16.22±0.8	17.25±1.1	0.52	0.48/0.24
Area of cytoplasm (μm^2^)	39.5±2.3	39.09±1.5	0.00	0.95/0.05	39.24±2.3	40.28±2	0.06	0.81/0.1
Nucleus/cytoplasm ratio	0.46±0.0	0.48±0.0	0.17	0.69/0.18	0.42±0.0	0.43±0.0	0.15	0.70/0.12
Area of mitochondria (μm^2^)	0.59±0.0	0.54±0.0	0.64	0.43/0.37	0.55±0.0	0.64±0.1	2.21	0.15/0.43
Vv of mitochondria (%)	5.3±0.9	3.9±0.7	1.13	0.30/0.45	4.07±0.4	3.12±0.4	2.08	0.16/0.48
*N* of mitochondria	3.62±0.6	2.7±0.4	1.08	0.31/0.45	2.98±0.3	2.28±0.4	2.09	0.16/0.45
Area of vacuoles (μm^2^)	1.01±0.5	1.21±0.3	0.01	0.91/0.19	2.42±0.7	1.02±0.2	4.72	0.05/0.7
Vv of vacuoles (%)	0.61±0.1	0.81±0.1	3.39	0.07/0.48	0.79±0.3	1.32±0.3	0.82	0.37/0.36
*N* of vacuoles	0.30±0.1	0.59±0.1	3.77	0.06/0.49	0.14±0.0	0.71±0.2	4.23	0.05/0.72
Area of lipofuscin granules (μm^2^)	1.6±0.3	1.57±0.2	0.01	0.92/0.03	1.63±0.3	1.96±0.3	0.19	0.67/0.31
Vv of lipofuscin granules (%)	0.77±0.4	1.27±0.3	1.08	0.31/0.36	0.56±0.2	1.68±0.5	5.04	0.03*/0.66
*N* of lipofuscin granules	0.18±0.1	0.32±0.1	2.14	0.12/0.49	0.12±0.0	0.43±0.1	5.71	0.02*/0.68
Cell density (N/mm^2^)	39.8±14.6	50.53±13.8	0.19	0.67/0.17	47.46±7.6	58.77±16.3	0.42	0.52/0.19

Estimates of the ultrastructural parameters and cell density (means ± S.E.M.) of oligodendrocytes adjacent to microglia, myelinated fibers, neurons, and capillaries in the control and in the SCZ groups and between-group differences (F, *p*-values, ANCOVA), Cohen’s *d*

*p < 0.05; **p < 0.01, ***p < 0.001

**Fig. 2:**
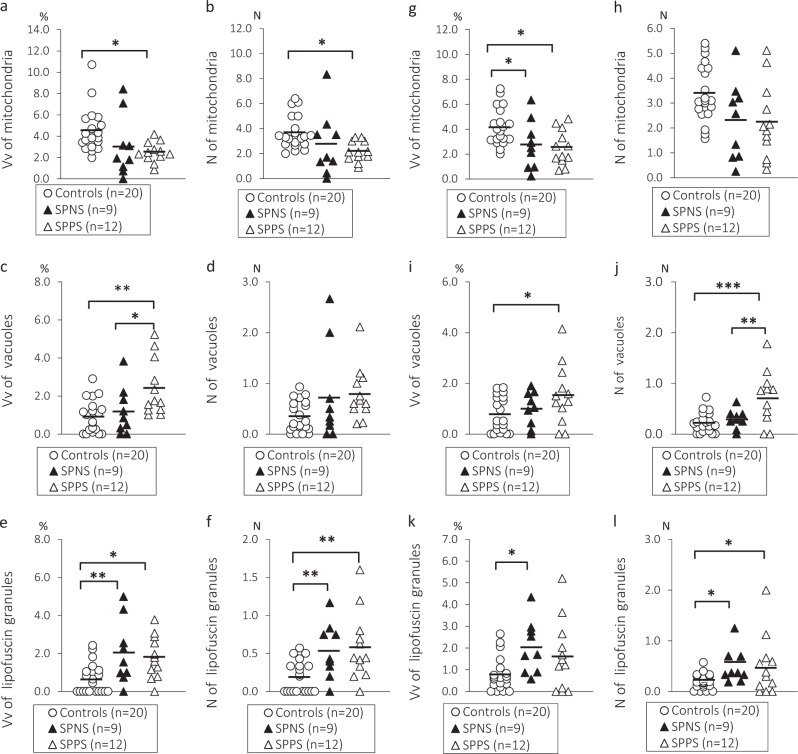
Individual mean values for Vv and *N* of mitochondria (**a**, **b**), vacuoles (**c**, **d**), lipofuscin granules (**e**, **f**) in oligodendrocytes adjacent to microglia, for the same parameters in oligodendrocytes adjacent to myelin (**g**–**l**) and the mean values for the control group, the subgroup with predominantly positive symptoms (SPPS) and the subgroup with predominantly negative symptoms (SPNS). *p < 0.05, **p < 0.01. ANCOVA and post-hoc Duncan test were used to determine the effect of clinical subgroups

A significant effect of clinical subgroups was found on Vv and *N* of mitochondria as well as on Vv and *N* of vacuoles and lipofuscin granules in oligodendrocytes adjacent to microglia (Table 3). Post-hoc Duncan test showed a significant decrease in Vv (45%, *p* < 0.05) and *N* of mitochondria (41%, *p* < 0.05) as well as a significant increase in Vv of vacuoles (160%, *p* < 0.01), lipofuscin granules (183%, *p* < 0.01) and *N* of lipofuscin (205%, *p* < 0.01) in the SPPS subgroup as compared to the control group. However, Vv and *N* of lipofuscin granules were also increased in the SPNS subgroup as compared to the control group (218% and 184%, respectively, *p* < 0.01) (post-hoc Duncan test). Vv of vacuoles was higher in the SPPS subgroup as compared to the SPNS subgroup (100%, *p* < 0.05) (post-hoc Duncan test). Vv of lipofuscin granules was higher in the SPNS subgroup as compared to the control group (218%, *p* < 0.05) (post-hoc Duncan test) (Fig. 2). A significant effect of clinical subgroups was also revealed on Vv of mitochondria as well as on Vv and *N* of vacuoles and lipofuscin granules in oligodendrocytes adjacent to myelinated fibers (Table 3). Post-hoc Duncan test showed a decrease in Vv of mitochondria (40%, *p* < 0.05), a significant increase in Vv of vacuoles (98%, *p* < 0.05), *N* of vacuoles (213%, *p* < 0.01), and *N* of lipofuscin granules (157%, *p* < 0.05) in the SPPS subgroup as compared to the control group. However, Vv and *N* of lipofuscin granules were also increased in the SPNS subgroup as compared to the control group (160%, *p* < 0.05) (post-hoc Duncan test). Effect size was largest in the SPPS subgroup for both oligodendrocytes adjacent to microglia and for oligodendrocytes adjacent to myelin (Table 3). *N* of vacuoles was higher in the SPPS subgroup as compared to the SPNS subgroup (60%, *p* < 0.01) (post-hoc Duncan test). Vv of mitochondria was lower (35%, *p* < 0.05) and *N* of lipofuscin granules was higher (160%, *p* < 0.05) in the SPNS subgroup as compared to the control group (post-hoc Duncan test) (Fig. 2). No effects of clinical subgroups on the parameters of peri-neuronal oligodendrocytes (*p* > 0.19), peri-capillary oligodendrocytes (*p* > 0.07), or on cell density of all subtypes of oligodendrocytes were found (*p* > 0.18) (ANCOVA).

**Table 3:** Effect of clinical subgroups on oligodendrocyted adjacent to microglia and myelin

	Oligodendrocytes adjacent to microglia						
	Controls (*n*=20)	SPNS (*n*=9)	SPPS (*n*=12)	F(2,36)	*p*-value	Cohen’s *d* SPNS/Controls	Cohen’s *d* SPPS/Controls
Area of oligodendrocyte (μm^2^)	61.51±2.6	62.59±3.6	64.3±4.3	0.36	0.70	0.09	0.22
Area of nucleus (μm^2^)	18.16±1.0	17.54±1.4	19.11±1.3	0.57	0.57	0.14	0.21
Area of cytoplasm (μm^2^)	43.35±2.0	45.05±2.6	45.19±3.1	0.29	0.75	0.19	0.19
Nucleus.cytoplasm ratio	0.43±0.0	0.39±0.0	0.43±0	0.52	0.60	0.33	0.06
Area of mitochondria (μm^2^)	0.51±0.0	0.5±0.0	0.5±0.0	0.08	0.92	0.21	0.17
Vv of mitochondria (%)	4.53±0.5	3.03±1.0	2.49±0.3	3.91	0.03*	**0.63**	**1.04**
*N* of mitochondria	3.7±0.3	2.76±0.9	2.19±0.2	3.39	0.04*	**0.52**	**1.10**
Area of vacuoles (μm^2^)	1.38±0.3	0.97±0.1	1.77±0.5	0.66	0.52	0.33	0.27
Vv of vacuoles (%)	0.92±0.2	1.18±0.4	2.38±0.4	6.92	<0.01**	**0.27**	**1.11**
*N* of vacuoles	0.35±0.1	0.72±0.3	0.78±0.2	3.20	0.05	0.63	0.97
Area of lipofuscin granules (μm^2^)	1.62±0.3	1.67±0.3	1.61±0.3	0.01	0.99	0.05	0.01
Vv of lipofuscin granules (%)	0.64±0.2	2.04±0.6	1.81±0.3	8.41	<0.01**	**1.08**	**1.08**
*N* of lipofuscin granules	0.19±0.0	0.54±0.1	0.58±0.1	8.01	<0.01**	**1.15**	**1.07**
Cell density (N/mm^2^)	69.04±9.9	53.33±11.1	64.88±7.0	0.49	0.62	0.38	0.11
	Oligodendrocytes adjacent to myelinated fibers						
	Controls (*n*=20)	SPNS (*n*=9)	SPPS (*n*=12)	F(2,36)	*p*-value	Cohen’s *d* SPNS/Controls	Cohen’s *d* SPPS/Controls
Area of oligodendrocyte (μm^2^)	63.43±2.4	64.48±3.0	65.48±3.2	0.11	0.89	0.10	0.19
Area of nucleus (μm^2^)	20.08±0.7	20.42±1.5	21.51±1.0	0.56	0.58	0.09	0.43
Area of cytoplasm (μm^2^)	43.34±1.9	44.06±2.2	43.97±2.3	0.02	0.98	0.09	0.07
Nucleus.cytoplasm ratio	0.47±0.0	0.47±0.0	0.49±0.0	0.35	0.71	0.01	0.36
Area of mitochondria (μm^2^)	0.53±0.0	0.5±0.0	0.59±0.1	0.57	0.57	0.36	0.27
Vv of mitochondria (%)	4.21±0.3	2.73±0.7	2.51±0.4	4.67	0.02*	**0.83**	**1.02**
*N* of mitochondria	3.42±0.3	2.29±0.5	2.23±0.4	3.26	0.05	0.81	0.84
Area of vacuoles (μm^2^)	1.67±0.3	1.55±0.3	1.11±0.2	0.83	0.44	0.12	0.55
Vv of vacuoles (%)	0.77±0.2	0.99±0.2	1.53±0.3	4.09	0.03*	**0.32**	**0.78**
*N* of vacuoles	0.22±0.0	0.29±0.1	0.69±0.1	11.53	<0.01***	**0.35**	**1.14**
Area of lipofuscin granules (μm^2^)	1.89±0.4	1.9±0.2	1.75±0.3	0.21	0.81	0.01	0.11
Vv of lipofuscin granules (%)	0.77±0.2	2.04±0.4	1.61±0.5	5.22	0.01*	**1.16**	**0.69**
*N* of lipofuscin granules	0.19±0.0	0.49±0.1	0.49±0.2	4.95	0.01*	**1.14**	**0.76**
Cell density (N/mm^2^)	144.2±22.0	122.5±18.5	84.45±18.5	1.76	0.19	0.25	0.65

Estimates of the ultrastructural parameters and cell density (means ± S.E.M.) of oligodendrocytes adjacent to microglial cells and to myelinated fibers in the control group and in the SCZ subgroups with predominantly positive symptoms (SPPS) and with predominantly negative symptoms (SPNS) and between subgroups and control group differences (F and *p*-values, ANCOVA), Cohen’s *d* for SPNS/controls and for SPPS/controls

*p < 0.05; **p < 0.01, ***p < 0.001

Comparison between oligodendrocytes adjacent to microglia and to myelin was performed using two two-way ANCOVA. In the first analysis the parameters of oligodendrocytes adjacent to microglia and of oligodendrocytes adjacent to myelin were used as dependent variables, diagnosis as independent factor, and age and post-mortem interval as covariates. In the secondary analysis the parameters of oligodendrocytes adjacent to microglia and of oligodendrocytes adjacent to myelin were used as dependent variables, clinical subgroups (SPPS, SPNS) as between-subjects factors, and age and post-mortem interval as covariates. Two two-way ANCOVA showed no significant differences between these two oligodendrocyte subpopulations (all *p* > 0.18). Lack of differences might be related to the large variance of the parameters measured in both subpopulations of oligodendrocytes. However, a comparison of the changes of the mean values of the parameters between these oligodendrocyte subpopulations in clinical subgroups as compared to the control group (expressed in %) showed decreased Vv of mitochondria (−5%), increased Vv of vacuoles (+62%), and increased Vv of lipofuscin granules (+76%) in oligodendrocytes adjacent to microglia as compared to oligodendrocytes adjacent to myelin in the SPPS subgroup. Effect size was largest for the parameters of oligodendrocytes adjacent to microglia in the SPPS subgroup (Table 3).

**Fig. 3:**
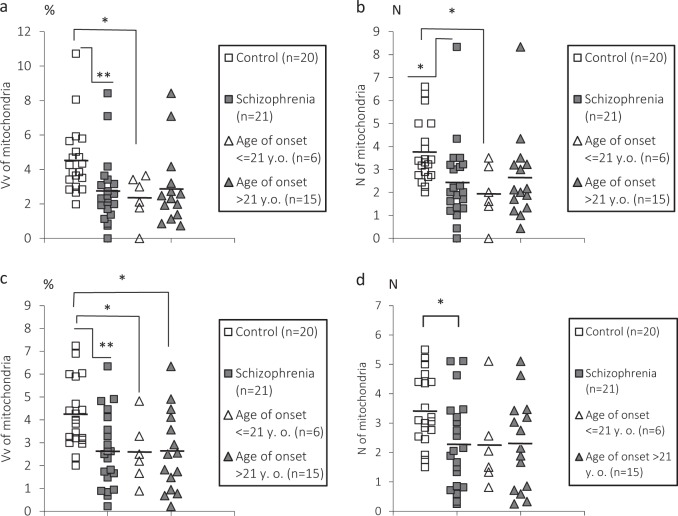
Individual mean values for Vv and *N* of mitochondria in oligodendrocytes adjacent to microglia (**a**, **b**), in oligodendrocytes adjacent to myelin (**c**, **d**), and the mean values in oligodendrocytes adjacent to microglia and to myelin for the control group and for the SCZ subgroups of age of disease onset <21 years old and >21 years old. *p < 0.05, **p < 0.01. ANCOVA and post-hoc Duncan test were used to determine the effect of age of onset of disease

We also found an effect of age of onset of disease on Vv of mitochondria: [F(2.37) = 3.73, *p* = 0.03] and *N* of mitochondria [F(2. 37) = 3.36, *p* = 0.04] in oligodendrocytes adjacent to microglia (ANCOVA). Post-hoc Duncan test showed a significant decrease in Vv and *N* of mitochondria (50%, *p* = 0.02) only in the subgroup of age of onset <21 years old as compared to the control group (Fig. 3a, b). In oligodendrocytes adjacent to myelinated fibers post-hoc Duncan test showed a significant decrease in Vv of mitochondria in the subgroup of age of onset <21 years old (40%) and in the subgroup of age of onset >21 years old (40%, *p* = 0.03) as compared to the control group (Fig. 3c, d). No effect of gender on the parameters measured was revealed (*p* > 0.1) (ANCOVA).

Pearson correlation analysis showed no effects of age and post-mortem interval on the parameters measured for both groups (*p* > 0.3). There were no significant correlations between the parameters measured and the duration of disease (*p* > 0.3). No significant correlations between oligodendrocyte parameters and chlorpromazine equivalents in the SCZ group were found (*p* > 0.2).

## Discussion

The present study showed prominent dystrophic alterations in oligodendrocytes adjacent to microglia and to myelinated fibers but not in oligodendrocytes adjacent to neurons or capillaries in WM of SCZ cases as compared to healthy controls. Mean Vv and *N* of mitochondria were significantly decreased while Vv of vacuoles of endoplasmic reticulum and of lipofuscin granules were significantly increased in oligodendrocytes adjacent to microglia and to myelin in the SCZ group and in the SPPS subgroup as compared to the control group. Oligodendrocyte cell density, areas of cell, nucleus, and cytoplasm were not changed in WM in SCZ. These data are in agreement with the results of Hercher et al.,^9^ who reported no changes in oligodendrocyte nuclear area in the prefrontal WM (BA9) in SCZ and with the data of Walker et al.^30^ who showed no changes in oligodendrocyte somata and nuclear areas in the substantia nigra in SCZ. No signs of degeneration were found in either SCZ or control brains. There were no significant effects of age, post-mortem interval or gender, as well as no significant correlations between the parameters measured and age, post-mortem delay, neuroleptic treatment or duration of illness. Thus, our data suggest that the dystrophic changes of oligodendrocytes in chronic SCZ patients are associated with the disease.

Our study demonstrated dystrophic alterations in oligodendrocytes adjacent to microglia similar to the changes of oligodendrocytes adjacent to myelin. We have found no significant group or subgroup differences (all *p* > 0.18) between oligodendrocytes adjacent to microglia and oligodendrocytes adjacent to myelin using ANCOVA. Thus, the data obtained provide no quantitative evidence that oligodendrocyte dystrophy is associated with microglial activation. The results of this study are difficult to interpret in the context of microglial activation because microglial cells were not included in morphometry. However, a comparison of the changes of the mean values of the parameters between these oligodendrocyte subpopulations in clinical subgroups as compared to the control group (expressed in %) showed lower Vv of mitochondria (−5%) and higher Vv of vacuoles (+62%) and lipofuscin granules (+76%) in oligodendrocytes adjacent to microglia compared to those adjacent to myelin in the SPPS subgroup. Effect size was largest for the parameters of oligodendrocytes adjacent to microglia in the SPPS subgroup (0.97–1.1). A significant reduction in Vv and *N* of mitochondria was found in the subgroup of SCZ patients with the age of onset <21 years old as compared to the control group (−50% in oligodendrocytes adjacent to microglia and −40% in oligodendrocytes adjacent to myelin). These data suggest that we cannot exclude the possibilities that microglia might strengthen dystrophic alterations of oligodendrocytes in the acute state of SCZ and that we would detect significant dystrophic alterations of oligodendrocytes adjacent to microglia as compared to oligodendrocytes adjacent to myelin in the SPPS subgroup using larger sample size. Since microglial cells are known to be dynamic, the similarity of the changes in oligodendrocytes adjacent to myelin with those adjacent to microglia might be associated at least in part with the effects of microglia during earlier phases of chronic SCZ.

Though we provide no quantitative evidence that oligodendrocyte dystrophy is associated with microglial activation, qualitative study gave some additional information. Some signs of activation of microglial cells adjacent to oligodendrocytes were seen in WM in the SCZ cases as compared to controls: the presence of microglia that contained enlarged cytoplasm, a nucleus of irregular contour and numerous vacuoles in cytoplasm; the presence of the contacts of microglial cytoplasmic membrane with oligodendrocyte nucleus found in three SCZ cases and in one control case in clustered microglia adjacent to clustered oligodendrocytes is a sign of microglial activation.^31^ In addition, some signs of possible toxic effects of microglia on oligodendrocytes were seen in the SCZ samples: focal lysis of cytoplasm of oligodendrocytes and the formation of large vacuoles in cytoplasm of oligodendrocytes located closely to contact between microglial cells and oligodendrocytes, dystrophic changes of oligodendrocytes adjacent to many ameboid and dystrophic microglial cells in the SPPS subgroup as compared to controls. Previously we have reported a significant increase in areas of the nucleus and cytoplasm of microglial cells adjacent to oligodendrocytes in the prefrontal WM in the SPPS subgroup as compared to controls.^28^ Significant dystrophic changes of oligodendrocytes adjacent to microglia in the SCZ group as compared to controls were found in the present study. Taken together, these data also do not exclude the possibility that microglial activation might contribute to dystrophic alterations of oligodendrocytes in WM in SCZ. These data are in line with some evidence for microglial activation in SCZ: the associations between the magnitude of microglia ligand binding and SCZ symptom severity,^24,25^ increased protein and mRNA levels of TNF-α in the prefrontal cortex,^32^ proinflammatory cytokines and HLA-DP/Q/DR expression correlated with the expression of IL-1 in the prefrontal cortex in SCZ.^17^ However, it is important to note that such confounders like autoimmunity, pneumonia, other viral or bacterial infection, obesity, metabolic syndrome could potentially impact microglia activation.^33^

We found no effects of neuroleptic drugs on the parameters measured. Antipsychotics are known to exert a beneficial influence on both microglia and oligodendrocytes. The inhibitory effects of some typical and/or atypical antipsychotics on the release of inflammatory cytokines and free radicals from activated microglia have been reported.^34^ Quetiapine mitigates the neuroinflammation and oligodendrocyte loss in the brain of C57BL/6 mouse following cuprizone exposure for one week.^35^ Pretreatment of aripiprazole and minocycline, but not haloperidol, suppresses oligodendrocyte damage from interferon-γ-stimulated microglia in a co-culture model. ^36^ Anti-inflammatory treatment with cyclooxygenase-2 (COX-2) inhibitors have shown beneficial effects in SCZ patients by blocking the synthesis of proinflammatory prostaglandins.^37^ However, Cotel et al.^38^ showed that chronic antipsychotic medication for 8 weeks to adult rats increased density of total microglia and specifically of ameboid microglia in different brain regions. But these data were obtained in normal rats. Taken together these data suggest that the dystrophic changes of oligodendrocytes detected in the present study are not attributable to antipsychotic treatment.

Oligodendrocytes are highly vulnerable to inflammation, hypoxia–ischemia, oxidative stress, and elevated glutamate levels that lead to excitotoxicity.^2^ The results of WM MRS study have shown that acute psychosis^39^ and SCZ in elderly patients^40^ were both associated with higher glutamate levels that lead to excitotoxicity. SCZ has also been associated with oxidative stress and chronic inflammation, both of which appear to reciprocally induce each other in a positive feedback manner.^27^ Genetic studies have shown associations between oxidative stress gene polymorphisms and SCZ. Increased oxidative stress and oxidative DNA damage have been reported in non-remission patients with SCZ.^41^ In response to stress, oligodendrocytes produce several immune mediators known to modulate the activation state of microglia. One mechanism by which TNF-α and IL-1β contribute to oligodendrocyte damage is via the inducible isoform of the nitric oxide synthetase gene activation leading to NO production that induces cytolysis and inhibition of the Kreb’s cycle and oxidization, via reactive oxygen species (ROS) production in oligodendrocytes.^27^ Total antioxidant and glutathione plasma levels are lower in non-medicated, medicated, first-episode as well as chronic SCZ patients, the reduced glutathione levels found in the prefrontal cortex in patients in which abnormal redox-related protein expression has also been found.^42^ Mitochondria are major sources of ROS; mitochondrial malfunction can lead to cellular degeneration because of the formation of ROS.^43^ Reduced Vv and the number of mitochondria found in the present study is consistent with alterations in mitochondrial energy metabolism and oxidative stress, including the prefrontal cortex.^44^

We found the effect of age of onset of disease on oligodendrocytes adjacent to microglial cells and to myelinated fibers (a significant reduction in Vv and the number of mitochondria were found in the subgroup of SCZ patients with age of onset <21 years old as compared to the control group). The data agree with the hypothesis of Maas et al.^42^ that oxidative stress during late adolescence impairs oligodendrocyte precursor signal transduction processes that are necessary for their proliferation and differentiation. Attenuation of proliferation in oligodendrocyte precursor cells by activated microglia has been reported.^45^ The data suggest that the deficit of oligodendrocyte density in the prefrontal WM previously reported^5,6^ might be associated with microglial activation at the early stage of SCZ. Goudriaan et al.^46^ have shown that astrocyte and oligodendrocyte gene sets, but not microglia gene sets, are associated with an increased risk for SCZ, oligodendrocyte lipid metabolism, and oxidation–reduction. These data suggest that microglial reactivity in WM in SCZ might be secondary to the disturbance of oligodendrocyte metabolism.

### Limitations

Our study has some limitations. First, the present study was limited by a small number of oligodendrocytes adjacent to microglia (7 cells per case) and small subgroup sizes. Second, most of the subjects in our study were older than 55 years old with long duration of disease (average 26 years). Third, the effects of neuroleptic treatment remain uncertain. Fourth, the estimation of predominantly positive and predominantly negative symptoms was derived from two separate scales used at the time of autopsy. Fifth, we found clustered microglia adjacent to clustered oligodendrocytes and the contacts of the microglial cytoplasmic membrane with the nuclei of oligodendrocytes in three SCZ cases and in one control case as a sign of microglial activation. These data should be replicated and be of interest for quantitative studies. Sixth, the morphometry of the ultrastructural parameters of microglia is required to clear up whether microglia is activated and/or dystrophic in SCZ.

## Conclusion

In conclusion, this study provides no quantitative evidence that oligodendrocyte dystrophy is associated with microglial activation in prefrontal WM in SCZ. Oligodendrocyte dystrophy was most pronounced in the SCZ subjects with predominantly positive symptoms as compared to healthy controls. The presence of many ameboid and dystrophic microglia adjacent to oligodendrocytes and significant dystrophic changes of oligodendrocytes adjacent to microglia in SCZ as compared to controls do not exclude the possibility that microglial activation might contribute to dystrophic alterations in prefrontal WM in SCZ. Future studies of the role of microglia in oligodendrocyte pathology in SCZ and the relation to clinical symptoms are needed to consider these cells as a target for new treatment strategy of SCZ.

## Methods

### Subjects

Twenty-one cases with SCZ and 20 normal controls were used for the study. Post-mortem brain tissue was obtained from the Anatomical Department of Moscow Psychiatric Hospitals No. 1 and No. 15 and Moscow Higher Medical School. ICD-10 and DSM-IV-R diagnostic criteria were used by psychiatrists. Independent psychiatrists evaluated medical records using the Scale for the Assessment of Negative Symptoms (SANS) and the Scale for the Assessment of Positive Symptoms (SAPS) to rate negative and positive symptoms during the last hospitalization in SCZ subjects. The predominantly positive or the predominantly negative symptoms were estimated on the basis of some integrative characteristics obtained including common score of positive and negative symptoms (in points), relative frequency (%) of positive and negative symptoms, and relative frequency (%) of the most severe positive and negative symptoms. Basic demographic and clinical data are given in Table 1. Cases were coded for morphometric blind study. After receiving сonsent for autopsy and research and approval for the study from the Ethics Committee of Mental Health Research Center, samples of the WM underlying layer VI of the frontal lobe (Brodmann’s area 10) from the left hemisphere were dissected from the brains. Data on age of onset, duration of illness, and neuroleptic exposure were taken from medical records. Medical records were analyzed and chlorpromazine equivalents were estimated for the patient's last 30 days. Causes of death were the same in the control and the SCZ groups: heart failure, pneumonia, pulmonary embolism, myocardial infarction, chronic cardiovascular disease, respiratory failure, cardiac disease, cardiac arrest, aneurisma. Cancer, cases of comorbid alcoholism, or drug abuse were excluded.

### Tissue preparation

Tissue specimens containing WM were taken from each case and fixed by immersion with a mixture of 2.5% glutaraldehyde and 4% paraformaldehyde in 0.1 M phosphate buffer (pH = 7.4) for one week. Then they were dissected out of the larger fixed tissue samples into 10 small tissue pieces for each case, washed in phosphate buffer (pH = 7.4), postfixed in 2% osmium tetroxide, and embedded in Araldite epoxy resin blocks. Semi-thin (1 μm) sections were collected on glass slides and stained with 1% methylene blue and used to choose the area on the blocks before trimming for ultrathin sectioning. A small pyramid was trimmed from two randomly selected tissue blocks for each case (among 10 blocks prepared for each case). Ultrathin (60 nm) sections were cut using Reichert ultramicrotome, collected on carbon-stabilized, formvar-coated copper slot grids, followed by staining with uranyl acetate and lead citrate. Sections were examined with a JEM-100B (JEOL, Japan) electron microscope. Electron micrographs of oligodendrocytes were taken at ×3300 magnification. In electron microscopic preparations oligodendrocytes contain short cisternae of granular endoplasmic reticulum, polyribosomes, rather short mitochondria, and profiles of the Golgi apparatus. The profiles of nuclei of oligodendrocytes are generally round to oval. Microglial cell bodies generally had elongated nuclei but some nuclei had either round or triangular shaped nuclei with electron-dense heterochromatin, and the center of these nuclei had lighter chromatin. Cytoplasm of these cells contains mitochondria, cisternae of endoplasmic reticulum, Golgi complexes, lysosomes, or lipofuscin granules.^47^

### Morphometry

Morphometry of oligodendrocytes adjacent to microglia and of oligodendrocytes adjacent to myelinated fibers, neurons, and capillaries that were not adjacent to microglia was performed. Mean oligodendrocyte cell number per case collected ± S.D. were: 7.1 ± 3.1 of oligodendrocytes adjacent to microglia for the control group and 7.2 ± 2.8 for the SCZ group; 15.0 ± 7.2 of oligodendrocytes adjacent to myelinated fibers for the control group and 12.5 ± 7.6 for the SCZ group; 4.6 ± 4.2 of peri-neuronal oligodendrocytes for the control group and 6.6 ± 4.9 for the SCZ group; 5.0 ± 2.3 of peri-capillary oligodendrocytes for the control group and 6.1 ± 5.1 for the SCZ group. Oligodendrocytes were counted within the area (mean ± S.D) 0.25 ± 0.05 mm^2^ in the control group and 0.26 ± 0.05 mm^2^ in the SCZ group. Cell density of the counted oligodendrocytes was estimated as the number of cells per unit tissue area (N/mm^2^). Oligodendrocyte size (areas of cell, nucleus, and cytoplasm), nucleus/cytoplasm ratio, volume density (Vv) and the number (*N*) of organelles (mitochondria, vacuoles of endoplasmic reticulum, and dense lipofuscin granules) were measured. Areas of oligodendrocytes adjacent to microglial cells, myelin, neurons and capillaries, of their nucleus, cytoplasm and of organelles were estimated using test grids for two-dimensional counts, superimposed on the negatives at the final magnification ×26,000. The interpoint distance on the test grid was equal to 1 µm for cells and 0.2 μm for organelles. Volume fraction (Vv, %) of cytoplasmic organelles were estimated according to the “Delesse principle”: Vv = Aa, i.e., measurements of the cross-sectional area of organelles relative to the cross-sectional area of cytoplasm give unbiased estimate of volume fraction of the objects.^48^

### Statistical analysis

Statistical analysis was performed using Statistica software, version 7 (Stat Soft). The significance threshold <0.05 was used. The data obtained were examined using the Kolmogorov–Smirnov test for normality. Since quantitative data were normally distributed, a Pearson correlation analysis was performed to assess possible correlations between the parameters measured and age, post-mortem interval, treatment with antipsychotic drugs (CPZ equivalents), and duration of disease. The groups did not differ significantly by age (ANOVA *p* = 0.9) and post-mortem delay (*p* = 0.2). Comparisons between the SCZ patients and controls were performed using analysis of covariance (ANCOVA) with oligodendrocyte parameters measured as dependent variables, diagnosis as independent factor, and age and post-mortem interval as covariates. To determine the effect of clinical SPPS and SPNS subgroups on the parameters measured, we used ANCOVA with oligodendrocyte parameters as dependent variables, clinical SPPS and SPNS subgroups as between-subjects factors, and age and post-mortem interval as covariates. To determine the effect of gender on the parameters measured, we used ANCOVA with oligodendrocyte parameters as dependent variables, diagnosis and gender as between-subjects factors, and age and post-mortem interval as covariates. Following ANCOVA, a post-hoc Duncan test was performed. Effect size was estimated using Cohen's *d* in the Statistica software. Some parameters in the SCZ group and in clinical subgroups had outliers. However, there were no outliers in other parameters in these cases, so the cases that had outliers were not excluded from the statistical analysis.

## Data Availability

Data available on request from the authors.
